# Overexpression of GRK3, Promoting Tumor Proliferation, Is Predictive of Poor Prognosis in Colon Cancer

**DOI:** 10.1155/2017/1202710

**Published:** 2017-12-28

**Authors:** Tao Jiang, Chun Yang, Liyuan Ma, Zehua Wu, Ling Ye, Xiaoqiang Ma, Hai Li, Junwei Fan, Yinxue Yang

**Affiliations:** ^1^Department of Anal-Colorectal Surgery, General Hospital of Ningxia Medical University, 804 South Shengli Road, Yinchuan 750004, China; ^2^Department of Ultrasound, General Hospital of Ningxia Medical University, 804 South Shengli Road, Yinchuan 750004, China; ^3^Department of Hepatobiliary Surgery, the Affiliated Hospital of Medical College, Qingdao University, Qingdao 266000, China; ^4^Department of General Surgery, Shanghai Jiao Tong University Affiliated First People's Hospital, 85 Wujin Road, Shanghai 200080, China

## Abstract

Deregulation of G protein-coupled receptor kinase 3 (GRK3), which belongs to a subfamily of kinases called GRKs, acts as a promoter mechanism in some cancer types. Our study found that GRK3 was significantly overexpressed in 162 pairs of colon cancer tissues than in the matched noncancerous mucosa (*P* < 0.01). Based on immunohistochemistry staining of TMAs, GRK3 was dramatically stained positive in primary colon cancer (130/180, 72.22%), whereas it was detected minimally or negative in paired normal mucosa specimens (50/180, 27.78%). Overexpression of GRK3 was closely correlated with AJCC stage (*P* = 0.001), depth of tumor invasion (*P* < 0.001), lymph node involvement (*P* = 0.004), distant metastasis (*P* = 0.016), and histologic differentiation (*P* = 0.004). Overexpression of GRK3 is an independent prognostic indicator that correlates with poor survival in colon cancer patients. Consistent with this, downregulation of GRK3 exhibited decreased cell growth index, reduction in colony formation ability, elevated cell apoptosis rate, and impaired colon tumorigenicity in a xenograft model. Hence, a specific overexpression of GRK3 was observed in colon cancer, GRK3 potentially contributing to progression by mediating cancer cell proliferation and functions as a poor prognostic indicator in colon cancer and potentially represent a novel therapeutic target for the disease.

## 1. Introduction

Colon cancer is the third most common cancer and the fourth cause of cancer mortality globally [1, 2]. Although the prognosis was steadily or started to increase by strategy for standard curative resection-based, multidisciplinary, and comprehensive therapy of colon cancer, the 5-year relative survival remains discouraging especially in low-income countries [3]. The molecular pathogenesis of colon cancer is heterogeneous including the accumulation of genetic and epigenetic changes, which are clinically important because they are related to the prognosis and treatment response of the patients [4]. Metastasis and resultant organ failure are the leading cause of death for cancer patients; however, the molecular pathogenesis that regulates primary tumor to the metastatic phenotype is currently not well known. Therefore, novel prognostic biomarkers and target-specific therapies need to be identified for developing further improved treatment strategy.

G protein-coupled receptor kinase 3 (GRK3), also known as *β*-adrenergic receptor kinase 2, belongs to a subfamily of kinases called GRKs [5, 6]. GRK3 is best known to specifically phosphorylate the agonist-occupied form of the *β*-adrenergic and related G protein-coupled receptors, leading to broadly regulate receptor function [7, 8]. Previous reports showed that the aberrant overexpression of GRK3 acts as a promoter mechanism in some kinds of tumors, including prostate cancer and breast cancer, especially in metastasis [9–11]. GRK3 also has been shown a negative regulator of cell growth in a subtype of glioblastoma [12], suggesting a subtype and tissue-specific role of GRK3, which may result from tumorigenic pathways or tumor microenvironment in different cancer types. Here, we examine the GRK3 expression patterns and clarify the pathological significance and patient survival in colon cancer. Then, we demonstrate that GRK3 is essential for survival and proliferation in vitro and in vivo. Essential kinases associated with colon cancer pathogenesis need to be identified which may eventually lead to the discovery of novel drug targets for colon cancer.

## 2. Material and Methods

### 2.1. Tissue Samples and Patient Information

162 tissue samples from patients with biopsy-proven colon cancer were obtained fresh at the time of surgery and snap frozen in liquid nitrogen until quantitative real-time PCR analysis. In each case, paired tumor and uninvolved proximal mucosa were collected. All patients provided informed consent, and the study was approved by the Institutional Research Ethics Committee. In addition, a total of 180 paraffin-embedded tissue samples from two independent tissue microarray, TMA (90 cases of colon cancer tissues paired with normal mucosa purchased from Outdo Biotech, Shanghai, PR China), was prepared for histological studies and immunostaining analysis. None of the patients had received radiotherapy or chemotherapy before surgery, and the pathologic verification of diagnosis and staging was summarized according to the National Comprehensive Cancer Network (NCCN) practice guidelines. The follow-up of this cohort ended in August 16, 2015, and the median duration of follow-up was 47 (range, 3–73) months. Overall survival (OS) and disease-free survival (DFS) rates were defined as the interval from the initial surgery to clinically or radiologically proven recurrence/metastasis and death, respectively.

### 2.2. RNA Extraction and Quantitative Real-Time PCR

Total RNA extraction of 162 pairs of frozen specimens or cultured colon cancer cells was performed according to the manufacturer's instructions (AllPrep DNA/RNA Mini Kit, Qiagen, Germany). The RNA concentration and purity were measured by NanoDrop 2000 UV-vis spectrophotometer (Thermo Scientific, USA). First-strand cDNA was synthesized from one microgram of total RNA using the A3500 RT-PCR System (Promega, USA). Quantitative real-time PCR was performed on a Mastercycler® ep realplex (Eppendorf, Germany) with a SYBR Green RNA PCR kit (Fermentas, USA) according to the manufacturer's protocol. GRK3 was amplified with the following primers: 5′-gcagtgccgactggttct-3′ (forward primer) and 5′-gtctgaaagggctgtgacct-3′ (reverse primer); *β*-actin was used as an internal control with the following primers: 5′-cgggaaatgtgcgtgac-3′ (forward primer) and 5′-tggaaggtggacagcgagg-3′ (reverse primer). Each reaction was run in triplicate. The relative GRK3 mRNA expression was calculated using 2^−△△Ct^ comparative method.

### 2.3. Western Blot Analysis

Total protein of cultured cells was extracted and measured using the BCA protein assay kit (Beyotime, China). Each 50 *μ*g aliquot of total protein was separated in 8% SDS-PAGE gel and then transferred onto polyvinylidene difluoride membranes (Millipore, Billerica, MA). The membranes were blocked in 5% skim milk for 1 hour at room temperature and then incubated overnight with the appropriate primary rabbit antibody against human GRK3 protein (1 : 1000 dilution, Abcam, United Kingdom) and *β*-actin (1 : 1000 dilution, Abcam, United Kingdom). Following incubation with a horseradish peroxidase-conjugated goat anti-rabbit secondary antibody (1 : 5000 dilution, Santa Cruz, USA), the bands were visualized by using the ECL Plus enhanced chemiluminescence kit (Pierce Biotechnology, USA) and exposure to Syngene GBOX/iCHE gel imaging and analysis systems. The *β*-actin expression used to confirm and normalize equal loading of the samples. Each sample was loaded in triplicate.

### 2.4. Immunohistochemistry on TMA

Tissue microarray was purchased from Shanghai Outdo Biotech Company Limited. Sections (4 *μ*m) of TMA slides were processed for immunostaining using an Envision kit (Dako Cytomation, Denmark). The paraffin-embedded sections were dewaxed, rehydrated, and then antigen retrieved. Specimens were immunolabeled with anti-GRK3 antibody (Abcam, ab38294, United Kingdom, 1 : 200 dilution) overnight at 4°C and then incubated with goat anti-rabbit Envision System Plus-HRP (Dako Cytomation, Denmark). PBS was used as a negative control. The final immunoreactivity was evaluated by two independent pathologists in a blinded fashion according to the staining intensity and extent of staining. The staining intensity was graded as follows: 0 (negative staining), 1 (mild staining), 2 (moderate staining), and 3 (intense staining). The staining extent was scored using the following scale: 0 (no staining of cells), 1 (<25% of tissue stained positive), 2 (26%–50% stained positive), 3 (51–75% stained positive), and 4 (>75% stained positive). The sum of staining score (intensity plus extent) was defined as follows: 0–2, negative expression; 3-4, weak expression; and 5-6, strong expression.

### 2.5. Cell Culture

The human colon cancer cell lines including RKO, SW620, LoVo, and HT-29 were purchased from Type Culture Collection of the Chinese Academy of Science (Shanghai, China). The human normal epithelial cells NCM460 were obtained from INCELL (San Antonio, USA). All of the cells were maintained at 37°C in 5% CO_2_ in Dulbecco's modified Eagle's medium that was supplemented with 10% FBS.

### 2.6. RNA Interference

For downregulation of GRK3, a small hairpin RNA- (shRNA-) mediated RNAi target sequence was cloned into the pGCSIL-GFP vector according to the manufacturer's protocol. The GRK3-specific siRNA sequence was 5′-caagaaacaagugacaucaacucuu-3′. A scrambled shRNA sequence, 5′- uucuccgaacgagucacg-3′, was used as a negative control. Transfection of GRK3-shRNA or negative control-shRNA plasmid into colon cancer cell lines was performed by using Lipofectamine 2000 (Life Technologies, USA) according to the manufacturer's instructions. Stable transfected cell clones were selected in 2 *μ*g/ml puromycin-containing medium (Sigma, USA) and enriched by the limited dilution methods. Finally, the clone expressions of GRK3 were confirmed by qPCR and Western blot analysis.

### 2.7. Cell Proliferation and Colony Formation Assays

Exponentially growing cells were resuspended and seeded in 96-well plates at an initial density of 2 × 10^3^cells/well. At each time point, 10 *μ*l of CCK-8 solution was added in each well, and cells were incubated for 2 h at 37°C with 5% CO_2_. The absorbance at 450 nm was measured on the Gen5 microplate reader (BioTek, Winooski, VT, USA). For colony formation assay, 800 treated log-phase cells were seeded in 6-well plates. After 14-day culture, cells were fixed and stained by Giemsa solution. Colonies were counted and photographed. Each assay was performed in triplicate.

### 2.8. Flow Cytometry Cell Cycle and Apoptosis Assay

Cell cycle assays were conducted by using the FACSCalibur flow cytometer (BD Biosciences, USA). Briefly, collected cells (1 × 10^6^) were rinsed with PBS, fixed in 70% ethanol for 1 h at 4°C, and finally stained with 50 *μ*g/ml propidium iodide (Sigma, USA) staining solution containing 100 μg/ml RNAse. Cell apoptosis assay was conducted by using the FACSCalibur flow cytometer (BD Biosciences, USA). Briefly, cells (1 × 10^6^) were washed by binding buffer, then resuspended in staining buffer, and finally incubated with Annexin V-APC (eBioscience, USA) for 15 minutes. All experiments were performed in triplicate.

### 2.9. Xenografted Tumor Model

BALB/c male nude mice were maintained according to the guidelines that were established by the Shanghai Resource Center of Laboratory Animals of the Chinese Academy of Science. Tumors were generated by subcutaneously injecting 5 × 10^6^ RKO cancer cells with stably silenced GRK3 (sh-GRK3) or control cells. Tumor dimensions were measured once 5 days, and tumor volume was calculated using the following formula: width^2^ × length × 0.5. All mice were euthanized 21 days after inoculation, and their tumors were weighed.

### 2.10. Statistical Analysis

SPSS software 19.0 (SPSS, Chicago, IL, USA) was used for the analyses. All experiments were independently performed three times in triplicate. Unless otherwise stated, differences between the mean values were analyzed for significance using the two-tailed unpaired *t*-test. Survival curves were estimated using the Kaplan-Meier method, and the differences in survival rates between groups were compared by the log-rank test. Multivariate analyses were applied using Cox proportional hazards regression to investigate the independent factors predictive of patients' survival. Data are displayed as mean and standard deviation. *P* values of less than 0.05 were considered to be significant.

## 3. Results

### 3.1. Aberrant Upregulation of GRK3 in Colon Cancer

**Figure 1:**
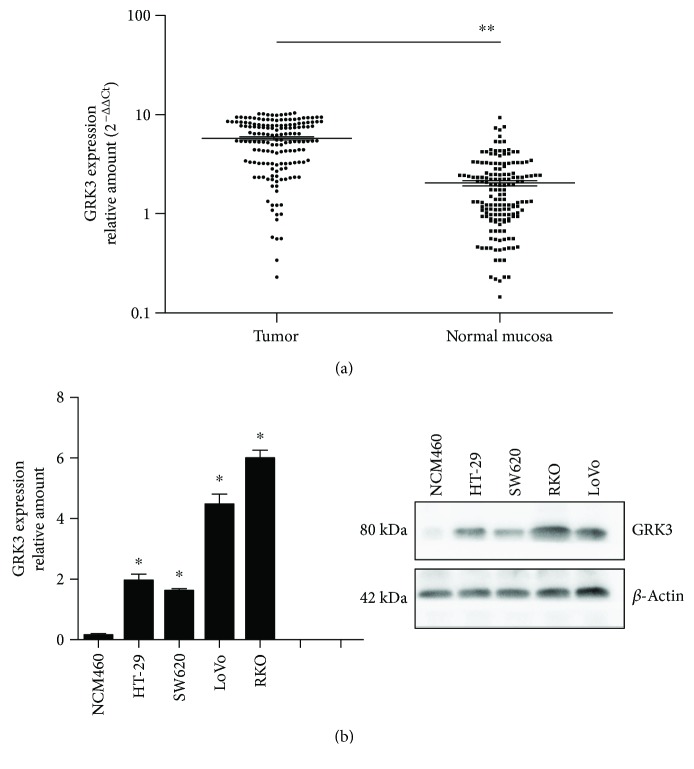
Analysis of GRK3 expression in tissues and cell lines of colon cancer. (a) Real-time quantitative polymerase chain reaction analysis of GRK3 expression in human colon cancer tissues and adjacent normal mucosa. Each relative GRK3 mRNA level was normalized using *β*-actin expression. ^∗∗^ *P* < 0.01 indicate statistical significance between two groups. (b) Real-time PCR (left) and Western blot analyses (right) were performed to investigate GRK3 expression in human colon cancer cell lines. Data are given as mean ± SD of three independent experiments. Statistical comparisons were made using two-tailed unpaired *t*-test. ^∗^ *P* < 0.05, GRK3 expression in different colon cancer cell lines versus NCM460 cells.

The expression patterns of GRK3 in colon cancer were confirmed by real-time PCR and Western blotting analyses in frozen colon cancer tissues and different cell lines. As shown in Figure 1(a), the relative level of GRK3 was significantly upregulated in 162 colon cancer tissues than in the matched noncancerous mucosa (*P* < 0.01). Furthermore, we explored GRK3 expression pattern in a panel of human colon cancer cell lines and normal colonic epithelium cells. Results indicated that GRK3 expression was markedly elevated in different colon cancer cell lines than in the normal colonic epithelium NCM460 cells (Figure 1(b)), which was identical to the results achieved from clinical specimens.

### 3.2. Association between GRK3 Expression and Clinicopathological Features of Colon Cancer

**Figure 2:**
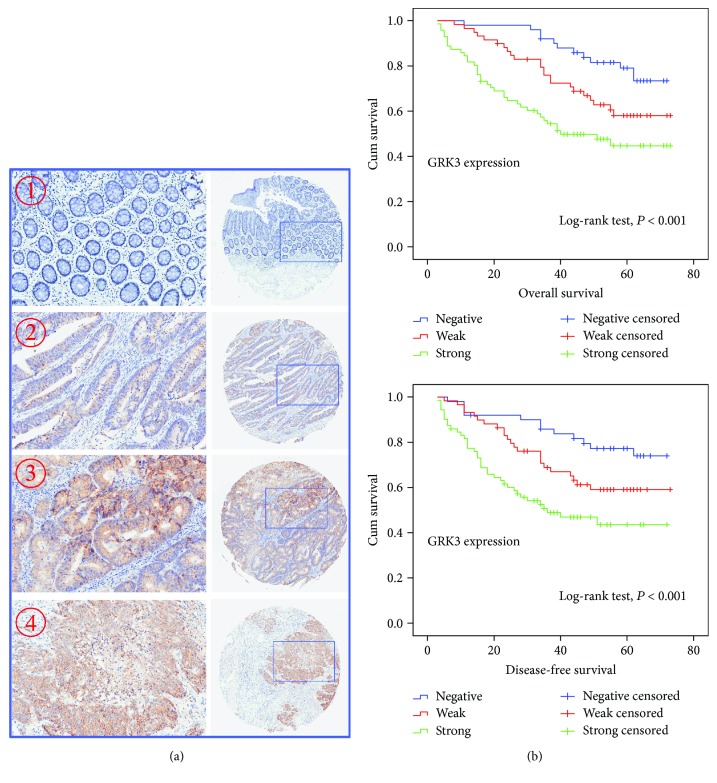
GRK3 expression and survival in colon cancer patients. (a) Immunohistochemical staining of GRK3 in tissue microarray (TMA). Elevated GRK3 expression in colon cancer tissues (2: well-differentiated case; 3: moderately differentiated case; and 4: low differentiated case) compared to adjacent normal mucosa with negative staining (1). (b) Kaplan-Meier survival curves of overall survival (top) and disease-free survival (bottom) according to GRK3 expression. Both overall survival rate and disease-free survival rate of GRK3-positive patients were significantly lower than those of the GRK3-negative patients (log-rank test, *P* < 0.001).

To further explore the association between GRK3 and clinical progression of colon cancer, the immunohistochemistry study was introduced to detect GRK3 expression in a total of 180 cases of primary colon cancer paired with noncancerous samples from two independent tissue microarray (TMA). The results of GRK3 antibody validation are shown in Supplementary Figure 1. Based on immunohistochemistry staining of TMAs, GRK3 was dramatically stained positive in primary colon cancer (130/180, 72.22%), whereas it was detected minimally or negative in paired normal mucosa specimens (50/180, 27.78%). The representative GRK3 expression pattern in both primary colon cancer and normal mucosa samples is shown in Figure 2(a). Of the 180 subjects, the correlation between GRK3 expression and clinicopathological characteristics was demonstrated in Table 1. We observed that the overexpression of GRK3 was closely correlated with American Joint Committee on Cancer Stage, AJCC (*P* = 0.001), depth of tumor invasion (*P* < 0.001), lymph node involvement (*P* = 0.004), distant metastasis (*P* = 0.016), and histologic differentiation (*P* = 0.004). No correlations were found between GRK3 expression and age, gender, or tumor location status. Collectively, all these results indicated that GRK3 may be involved and play a critical role in colon cancer carcinogenesis.

### 3.3. Overexpression of GRK3 Is an Independent Prognostic Indicator That Correlates with Poor Survival in Colon Cancer Patients

To assess the clinical value of GRK3 expression in colon cancer patients survival, Kaplan-Meier curves with a log-rank test for overall survival (OS) and disease-free survival (DFS) were undertaken. The 5-year OS rate of the 180 patients was 60%, and the 5-year DFS rate was 68.33%. As shown in Figure 2(b), patients with GRK3-positive (weak and strong) expression had a remarkably lower OS and DFS rate than patients with GRK3-negative expression (*P* < 0.05). The estimated mean OS time was significantly different between patients with GRK3-positive and GRK3-negative tumors (41.19 ± 20.105 and 56.26 ± 12.423 months, resp., *P* < 0.001). The estimated mean DFS time was 36.68 ± 19.785 and 52.04 ± 17.297 months for subjects with GRK3-positive and GRK3-negative tumors, respectively (*P* < 0.001). Furthermore, univariate and multivariate hazard ratios for survival were calculated by Cox proportional hazards model analyses. Univariate analyses indicated that AJCC stage, depth of tumor invasion, the presence of lymph node involvement, distant metastasis, histologic differentiation, and GRK3 expression pattern were significantly associated with DFS and OS (Tables 2 and 3). In addition, multivariate analyses were performed using the Cox proportional hazards model for all of the significant variables in the univariate analysis. Results revealed that GRK3 expression pattern was an independent prognostic indicator for the OS and DFS in colon cancer patients (hazard ratio: 0.424, 95% confidence interval: 0.226–0.797, *P* = 0.008 and hazard ratio: 0.453, 95% confidence interval: 0.241–0.854, *P* = 0.014, resp.) (Tables 2 and 3).

### 3.4. Downregulation of GRK3 Inhibits Proliferation of Colon Cancer Cells In Vitro

**Figure 3:**
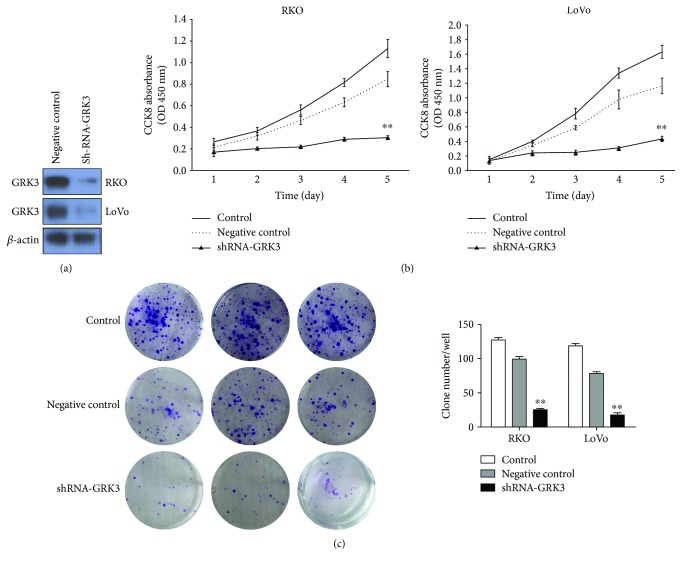
Downregulation of GRK3 inhibits proliferation of colon cancer cells in vitro. (a) Downregulation of GRK3 in different colon cancer cell lines was identified and confirmed by Western blot analysis. (b) The effect of GRK3 knockdown on different colon cancer cell growth was evaluated by CCK-8 assay (left: RKO cell line; right: LoVo cell line). Each point indicates the mean of the spectrometric absorbance ± SD of triplicate experiments. ^∗∗^ *P* < 0.01, shRNA-GRK3 versus negative control and control cells (two-tailed unpaired *t*-test). (c) Colony formation assay in different colon cancer cells. ^∗∗^ *P* < 0.01, shRNA-GRK3 versus negative control and control cells. Data are given as mean ± SD of three independent experiments. Statistical comparisons were made using two-tailed unpaired *t*-test. Representative RKO illustrations are shown in (c, left).

**Figure 4:**
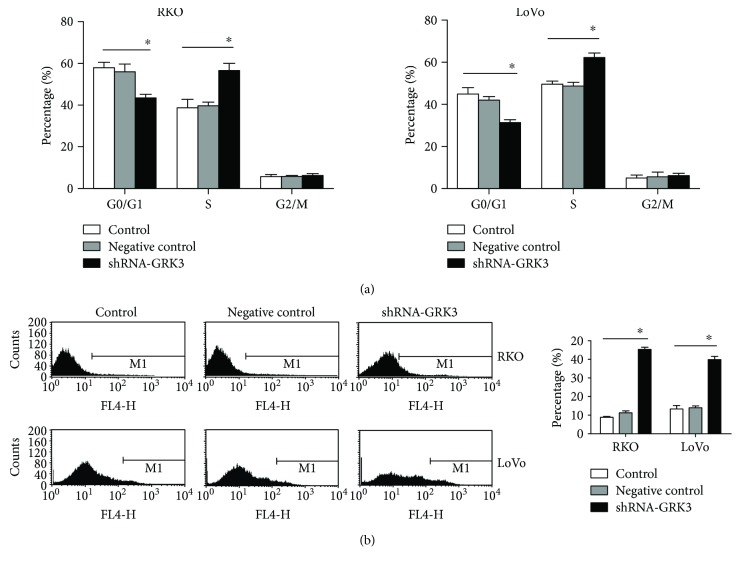
Downregulation of GRK3 increases S phase DNA content and induces apoptosis of colon cancer cells in vitro. Effect of GRK3 knockdown on different colon cancer cell apoptosis (a) and cell cycle (b) was examined by flow cytometry analysis. ^∗^ *P* < 0.05, shRNA-GRK3 versus negative control and control cells. Data are given as mean ± SD of three independent experiments. Statistical comparisons were made using two-tailed unpaired *t*-test. Representative illustrations are shown in (b, left).

To further determine the potential function of GRK3 in promoting colon cancer progression, we construct and identify a lentiviral vector harboring RNAi sequence targeting the human GRK3 and investigate the effects on a series of cancer-relevant in vitro cell-based assays testing proliferation, colony formation, cell cycle, and apoptosis. RKO and LoVo cells were transfected with shRNA-GRK3 or negative control-shRNA lentivirus, and stably transfected cells were established. Results indicated that expression of GRK3 was silenced by shRNA-GRK3 transfection in RKO and LoVo cell lines (Figure 3(a)). Cell Counting Kit-8 (CCK8) assays indicated that the proliferation of shRNA-GRK3 cells was significantly decreased as compared to the control groups in RKO and LoVo cells (*P* < 0.01, Figure 3(b)). These results demonstrate that GRK3 knockdown could inhibit the aggressive proliferation phenotype of colon cancer cells. In addition, downregulation of GRK3 consistently induced a reduction in colony formation ability (*P* < 0.01, Figure 3(c)). As shown in Figure 4(a), both RKO and LoVo cells lacking GRK3 accumulated increased S phase DNA content (S phase DNA content: 56.45 ± 3.51% versus 39.72 ± 1.56% and 62.13 ± 2.21% versus 48.64 ± 1.90% for RKO and LoVo cells, resp.). Moreover, the apoptosis rate was significantly elevated in both GRK3 knockdown cells compared with negative control cells (RKO: 11.29% to 45.23%, LoVo: 14.12% to 39.78%, Figure 4(b)). Collectively, these findings in vitro indicated that downregulation of GRK3 suppresses proliferation and induces apoptosis of colon cancer cells.

### 3.5. Downregulation of GRK3 Inhibits Tumor Growth In Vivo

**Figure 5:**
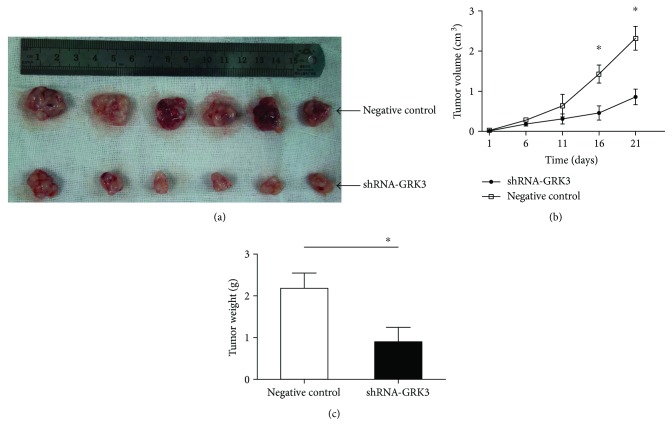
Downregulation of GRK3 expression inhibits tumorigenicity in vivo. (a) Stable GRK3 knockdown clones and control clones of RKO cells were injected subcutaneously into the flank of nude mice. (b) The tumor volume was calculated once 5 days. (c) All mice were euthanized 21 days after inoculation, and their tumors were weighed. Data are expressed as mean ± standard deviation. ^∗^ *P* < 0.05 indicate statistical significance between shRNA-GRK3 and the control group. Statistical comparisons were made using two-tailed unpaired *t*-test.

To evaluate the effect of GRK3 on tumorigenesis in vivo, RKO-shRNA-GRK3 or RKO-negative control cells were subcutaneously implanted in nude mice. Specifically, the average tumor size of shRNA-GRK3 tumors was reduced as compared to control cells at day 21 (0.86 ± 0.195 cm^3^ versus 2.32 ± 0.298 cm^3^, resp., *P* < 0.05, Figures 5(a) and 5(b)). Meanwhile, the average weight of shRNA-GRK3 tumors was reduced as compared to controls (0.90 ± 0.35 g versus 2.18 ± 0.37 g, resp., *P* < 0.05, Figures 5(a) and 5(c)). Taken together, GRK3 positively regulated cell proliferation in colon cancer in vivo models, which correlated with the results observed in the in vitro assays.

## 4. Discussion

Colon cancer is initiated by aberrant processing of genetic information due to genetic changes involving tumor suppressor genes and oncogenes, or altered epigenetic mechanisms manifested in global or local changes of chromatin structure [13, 14]. It is important to identify crucial genes that promote cancer progression. We report here a previously undescribed role of GRK3 in colon cancer. Our findings demonstrate the importance of GRK3 as a potential tumor progression promoter in colon cancer and its value as an independent prognostic marker of the disease. Significant associations were observed between aberrant GRK3 expression and advanced cancer biology, which was indicated by tumor invasion depth, lymph node metastasis, and distant metastasis. Moreover, we have demonstrated that downregulation of GRK3 is also sufficient to decrease primary tumor growth and induce apoptosis phenotype of importance. In addition, the results of *in vivo* studies were consistent with the results in vitro. Therefore, GRK3 is a key kinase and plays physiologic roles in the progression of human colon cancer.

GRK3 (also named *β*-adrenergic receptor kinase 2) belongs to the subfamily of G protein-coupled receptor kinases (GRKs) and is ubiquitously expressed in the body [8, 15]. GRK3 is generally known that specifically phosphorylates the agonist-occupied form of the beta-adrenergic and related G protein-coupled receptors, which may serve broadly to regulate receptor function [7, 15, 16]. GRK3 are highly expressed in different cellular types of the immune system and are important regulators of inflammation; the altered expression of GRK3 may play a pivotal role in cell motility in pathological situations related to inflammation or tumor progression [17–19]. Recently, several lines of evidence suggest that GRK3 involved to the oncogenic process, which indicates that the aberrant expression of GRK3 acts as a promoter mechanism in some kinds of tumors, including prostate and breast cancer [9, 11]. GRK3 contribute to angiogenesis and metastasis through downregulation of several genes involved in angiogenesis and microenvironment modulation, such as TSP-1 and PAI-2 in prostate cancer [9, 20, 21]. Also, it is intriguing that GRK3 not only controlled survival and proliferation of breast cancer cells but also promoted primary breast cancer invasion and metastasis by dysregulated signaling through CXCL12/CXCR4 [11]. However, little work has been done to explore the role of GRK3 in colon cancer.

Hallmarks of cancer are characterized by sustaining proliferative signaling, resisting cell death, enabling replicative immortality, inducing angiogenesis, and activating invasion and metastasis [4]. The complex biological processes correlated with alterations in genes related to regulation of proliferation, apoptosis, cell cycle, and genomic stability [22, 23]. Thus, identification and validation of the colon cancer-related target gene could promote further discovery of molecular mechanisms for colon cancer progression and provide new insights for predicting prognosis, therapeutic intervention, and development of novel tumor-targeting drugs in colon cancer. In the current study, GRK3 expression patterns were higher in colon cancer tissue compared with the paired adjacent normal mucosa. Based on immunohistochemistry staining of tissue microarray, GRK3 was dramatically stained positive in primary colon cancer; noncancerous mucosa specimens were minimally or negative for GRK3 expression. These findings suggested that GRK3 may be a novel colon cancer-related target gene and plays important role in the tumorigenesis. Our results revealed significant associations between the GRK3 overexpression and the following clinicopathological features: American Joint Committee on Cancer Stage, depth of tumor invasion, lymph node involvement, distant metastasis, and histologic differentiation. In addition, Kaplan-Meier curves with a log-rank test and Cox proportional hazards model analysis indicated that the patients with positive tumor GRK3 expression had a lower five-year overall survival and disease-free survival rate than patients with negative GRK3 expression. Multivariate analyses indicated that GRK3 expression in colon cancer was an independent prognostic factor for survival. Collectively, all these findings indicated that GRK3 involved colon cancer carcinogenesis and it could be possibly used as a biomarker to identify subsets of colon cancer with a more aggressive phenotype.

Kinases are known to be druggable, and several kinase inhibitors have been approved as cancer therapeutics [24–26]. Furthermore, the biology and functional study demonstrate that downregulation of GRK3 inhibited colon cancer cell proliferation rate in vitro and reduced tumorigenicity in vivo. In addition, we found that shRNA-mediated downregulation of GRK3 dramatically led to S-phase arrest and induce apoptosis phenotype of RKO and LoVo cells. GRK3 is a critical determinant of cellular responses to proliferative and migration signals through CXCL12/CXCR4 in breast cancer. Taken together, it is plausible that GRK3 functions as a cancer-promoting factor by promoting proliferation, although the molecular mechanisms need to be further elucidated.

In summary, our study shows that aberrant expression of GRK3 plays an important role in promoting colon cancer progression through enhanced proliferation and reduced apoptosis. GRK3 may be a clinical useful prognostic molecular biomarker for prognosis and a therapeutic target in colon cancer.

## Figures and Tables

**Table 1:** Associations of GRK3 expression with clinicopathological features in colon cancer (*n* = 180).

Variables	GRK3 expression			
	Negative (*n* = 50)	Weak (*n* = 59)	Strong (*n* = 71)	*P* Value^∗^
Age				
<65	25	20	32	*P* = 0.216
≥65	25	39	39	
Gender				
Male	29	38	37	*P* = 0.366
Female	21	21	34	
Location			
Right	15	19	30	*P* = 0.868
Transverse	7	8	9	
Left	6	8	8	
Sigmoid	22	24	24	
AJCC stage				
I	10	12	2	*P* = 0.001^∗^
II	26	20	27	
III	13	23	30	
IV	1	4	12	
T stage				
T1	3	3	0	*P* < 0.001^∗^
T2	7	10	3	
T3	38	37	40	
T4	2	9	28	
N stage				
N0	37	33	31	*P* = 0.004^∗^
N1	11	20	22	
N2	2	6	18	
M stage				
M0	49	55	59	*P* = 0.016^∗^
M1	1	4	12	
Differentiation				
Well	6	7	1	*P* = 0.004^∗^
Moderate	34	42	41	
Poor	10	10	29	

*P* values are based on Chi-square and Fisher's exact test. ^∗^Significant associations between 2 categorical variables.

**Table 2:** Cox proportional hazards model univariate and multivariate analyses of individual parameters for correlations with overall survival (OS).

Variable	Univariate analysis			Multivariate analysis		
	HR	CI (95%)	*P* value^∗^	HR	CI (95%)	*P* value^∗^
Age						
<65 years	1	—				
≥65 years	0.611	0.374–0.998	0.051			
Gender						
Male	1	—				
Female	1.070	0.667–1.714	0.780			
AJCC stage						
I and II	1	—		1	—	
III and IV	0.364	0.225–0.590	<0.001^∗^	0.458	0.277–0.756	0.002^∗^
T stage					NR	
T1 and T2	1	—				
T3 and T4	0.342	0.138–0.851	0.021^∗^			
Lymph node metastasis					NR	
Negative	1	—				
Positive	0.418	0.260–0.672	<0.001^∗^			
Distant metastasis					NR	
M0	1	—				
M1	0.144	0.075–0.276	<0.001^∗^			
Differentiation						
Well and moderate	1	—		1	—	
Poor	0.451	0.279–0.728	0.001^∗^	0.539	0.329–0.883	0.014^∗^
GRK3 expression						
Negative	1	—		1	—	
Weak and strong	0.375	0.201–0.698	0.002^∗^	0.424	0.226–0.797	0.008^∗^

HR: hazard radio; CI: confidence interval; NR: variable was not included in the resultant model. ^∗^Significance indicated that the 95% CI of HR was not including 1.

**Table 3:** Cox proportional hazards model univariate and multivariate analyses of individual parameters for correlations with disease-free survival (DFS).

Variable	Univariate analysis			Multivariate analysis		
	HR	CI (95%)	*P* value^∗^	HR	CI (95%)	*P* value^∗^
Age						
<65 years	1	—				
≥65 years	0.616	0.384–1.002	0.543			
Gender						
Male	1	—				
Female	1.065	0.665–1.707	0.793			
AJCC stage						
I and II	1	—		1	—	
III and IV	0.393	0.243–0.635	<0.001^∗^	0.496	0.301–0.819	0.006^∗^
T stage					NR	
T1 and T2	1	—				
T3 and T4	0.348	0.140–0.864	0.023^∗^			
Lymph node metastasis					NR	
Negative	1	—				
Positive	0.454	0.284–0.728	0.001^∗^			
Distant metastasis					NR	
M0	1	—				
M1	0.139	0.073–0.266	<0.001^∗^			
Differentiation						
Well and moderate	1	—		1	—	
Poor	0.478	0.297–0.772	0.003^∗^	0.577	0.352–0.945	0.029^∗^
GRK3 expression						
Negative	1	—		1	—	
Weak and strong	0.390	0.209–0.727	0.003^∗^	0.453	0.241–0.854	0.014^∗^

HR: hazard radio; CI: confidence interval; NR: variable was not included in the resultant model. ^∗^Significance indicated that the 95% CI of HR was not including 1.

## Supplementary Materials

Supplementary Materials Supplemental Figure 1: GRK3 antibody validation in colon cancer. Immunohistochemical staining of GRK3 on the same location of colon cancer tissues. (A, left) Anti-GRK3 antibody (Abcam, ab38294, United Kingdom) positive staining. (A, right) Negative control (PBS) negative staining. (B) GRK3 antibody validation experiments in SK-OV-3 and Mono-Mac-1 cells which represent for positive control and negative control, respectively.
