# Corrigendum to “Overexpression of GRK3, Promoting Tumor Proliferation, Is Predictive of Poor Prognosis in Colon Cancer”

**DOI:** 10.1155/2020/1245645

**Published:** 2020-10-14

**Authors:** Tao Jiang, Chun Yang, Liyuan Ma, Zehua Wu, Ling Ye, Xiaoqiang Ma, Hai Li, Junwei Fan, Yinxue Yang

**Affiliations:** ^1^Department of Anal-Colorectal Surgery, General Hospital of Ningxia Medical University, 804 South Shengli Road, Yinchuan 750004, China; ^2^Department of Ultrasound, General Hospital of Ningxia Medical University, 804 South Shengli Road, Yinchuan 750004, China; ^3^Department of Hepatobiliary Surgery, The Affiliated Hospital of Medical College, Qingdao University, Qingdao 266000, China; ^4^Department of General Surgery, Shanghai Jiao Tong University Affiliated First People's Hospital, 85 Wujin Road, Shanghai 200080, China

In the article titled “Overexpression of GRK3, Promoting Tumor Proliferation, Is Predictive of Poor Prognosis in Colon Cancer” [1], there was an error in the beta-actin forward primer. It was written as CGGGAAATGTGCGTGAC, and it should be corrected to CGGGAAATCGTGCGTGAC.
